# *Ixodes ricinus* ticks removed from humans in Northern Europe: seasonal pattern of infestation, attachment sites and duration of feeding

**DOI:** 10.1186/1756-3305-6-362

**Published:** 2013-12-20

**Authors:** Peter Wilhelmsson, Pontus Lindblom, Linda Fryland, Dag Nyman, Thomas GT Jaenson, Pia Forsberg, Per-Eric Lindgren

**Affiliations:** 1 Division of Medical Microbiology, Department of Clinical and Experimental Medicine, Linköping University, Linköping, Sweden; 2 Division of Infectious Diseases, Department of Clinical and Experimental Medicine, Linköping University, Linköping, Sweden; 3 The Åland Borrelia group, Mariehamn, Finland; 4 Medical Entomology Unit, Department of Systematic Biology, Evolutionary Biology Centre, Uppsala University, Uppsala, Sweden; 5 Clinic of Infectious Diseases, University Hospital, Linköping, Sweden; 6 Department of Microbiology, Ryhov County Hospital, Jönköping, Sweden

**Keywords:** *Ixodes ricinus*, Tick infestation, Tick bite, Attachment site, Feeding behaviour, Feeding duration, Host-seeking behaviour, Seasonal activity, Sweden, Åland

## Abstract

**Background:**

The common tick *Ixodes ricinus* is the main vector in Europe of the tick-borne encephalitis virus and of several species of the *Borrelia burgdorferi* sensu lato complex, which are the etiological agents of Lyme borreliosis. The risk to contract bites of *I. ricinus* is dependent on many factors including the behaviour of both ticks and people. The tick’s site of attachment on the human body and the duration of tick attachment may be of clinical importance. Data on *I. ricinus* ticks, which were found attached to the skin of people, were analysed regarding potentially stage-specific differences in location of attachment sites, duration of tick attachment (= feeding duration), seasonal and geographical distribution of tick infestation in relation to age and gender of the tick-infested hosts.

**Methods:**

During 2008–2009, 1770 tick-bitten persons from Sweden and the Åland Islands removed 2110 *I. ricinus* ticks. Participants provided information about the date of tick detection and location on their body of each attached tick. Ticks were identified to species and developmental stage. The feeding duration of each nymph and adult female tick was microscopically estimated based on the scutal and the coxal index.

**Results:**

In 2008, participants were tick-bitten from mid-May to mid-October and in 2009 from early April to early November. The infestation pattern of the nymphs was bimodal whereas that of the adult female ticks was unimodal with a peak in late summer. Tick attachment site on the human body was associated with stage of the tick and gender of the human host. Site of attachment seemed to influence the duration of tick feeding. Overall, 63% of nymphs and adult female ticks were detected and removed more than 24 hours after attachment. Older persons, compared to younger ones, and men, compared to women, removed “their” ticks after a longer period of tick attachment.

**Conclusions:**

The infestation behaviour of the different tick stages concerning where on the host’s body the ticks generally will attach and when such ticks generally will be detected and removed in relation to host age and gender, should be of value for the development of prophylactic methods against tick infestation and to provide relevant advice to people on how to avoid or reduce the risk of tick infestation.

## Background

The European tick *Ixodes ricinus* is a vector of several pathogens of humans and domesticated animals. The medically most important of these pathogens are the TBE virus (TBEV) causing tick-borne encephalitis (TBE) and the *Borrelia burgdorferi* sensu lato (s.l.) spirochaetes causing Lyme disease (= Lyme borreliosis, LB). *I. ricinus* is a three-host tick so to complete its life cycle, which consists of three active stages (larva, nymph and adult), it must ingest blood from a vertebrate host in each one of the three stages. Humans are, therefore, potential hosts for each one of these stages.

The northern limit of *I. ricinus* is determined by climate factors and access to suitable hosts [1,2]. In Sweden, the geographical distribution of *I. ricinus* covers the southern and central parts of the country as well as the coastal area of northern Sweden. Jaenson and co-workers suggested that a warmer climate with milder winters and a prolonged vegetation period have permitted important *I. ricinus* maintenance hosts, particularly roe deer (*Capreolus capreolus*), to spread to and inhabit previously climatically suboptimal areas in the northern parts of Sweden; this has resulted in a gradual spread northwards of *I. ricinus* infesting deer; in this manner the range and abundance of *I. ricinus* in northern Sweden increased considerably during the last 30 years [2]. Furthermore, the annual incidence of cases of neuroinvasive, human TBE increased significantly in Sweden during the years 2000–2012. This was presumably partly a consequence of the high tick abundance [3]. On most islands of the Åland archipelago (the Åland Islands), located in the Baltic Sea between Sweden and the mainland of Finland, *I. ricinus* is abundant and tick bites are commonly reported by the inhabitants [4].

The seasonal host-seeking activity pattern of *I. ricinus* is quite variable and not yet fully understood. However, it is influenced by several biotic and abiotic factors including vegetation type, density and variety of hosts, weather and climate, and the photoperiod (which is dependent on latitude) [5,6]. In two investigations conducted in south-central Sweden, nymphs and larvae of *I. ricinus* usually exhibited bimodal host-seeking activity patterns with the highest activity in May-June and August-September, and a midsummer activity depression [7,8]. It is proposed that the midsummer depression in host-seeking activity of subadult ticks may partly be due to the relatively dry conditions that usually prevail at this time [7]. During such a reduction in host-seeking activity, one would expect a lower tick infestation on animals and humans. In contrast to nymphs, adult ticks exhibited a unimodal host-seeking pattern without any midsummer depression [7]. From a medical point of view it is certainly relevant to study the tick infestation pattern on humans. This risk of tick infestation is dependent on the behaviour of both ticks and humans, which are influenced by weather conditions, climate and other factors.

Berglund and co-workers carried out an extensive epidemiological study of LB in southernmost Sweden [9]. They recorded a significantly greater proportion (20%) of neurological manifestations among LB patients who had been tick-bitten on the head or neck, than among LB patients bitten on other parts of their body (7%). Therefore, the tick’s “preferred” site for attachment on the human body may be of clinical importance. Attachment sites “preferred” by *I. scapularis* ticks, the main vector of LB spirochaetes in north-eastern United States, are, to a certain extent, dependent on the developmental stage of the tick: adult females of *I. scapularis* attach more frequently to the head and neck area, than to other parts of the human body [10]. Such a biting behaviour has, to our knowledge, not been reported for *I. ricinus* adult females that bite humans. However, “preferred” attachment sites on sheep are influenced by the developmental stage of *I. ricinus*: larvae attach mainly to the lower parts of the body and adult females mainly to the upper parts, while nymphs will attach mainly to sites in between those of larvae and adults [11]. Berglund and co-workers also recorded tick bites more often on the head and neck area of children with LB compared to the same body region of adults with LB [9]. This could suggest fundamental differences in how ticks respond to hosts of different sizes and/or ages. It may also suggest that transmission of the *Borrelia* spirochaetes is more efficient when the tick is attached to a particular body region.

The duration of tick feeding, including salivation and blood ingestion, is important for transmission of pathogens. The virions of TBEV-infected ticks will begin to be transmitted with the tick’s saliva to the vertebrate host almost instantaneously after tick attachment (< 1 hour) [12]. In contrast, *Borrelia burgdorferi* s.l. spirochaetes are not transmitted immediately, but the risk increases with the duration of tick feeding [13,14]. The risk may, in fact, already be present during the first 24 hours of tick feeding [14]. In general, however, tick-bitten people that remove ticks later than 24 hours of tick attachment are more likely to develop localised and systemic symptoms [15], probably due to injected tick salivary gland proteins and/or due to transmitted pathogens. Falco and co-workers reported that people bitten by adult female ticks of *I. scapularis* in North America were detected and removed earlier than nymphs [10]. They also reported that nymphal attachment times increased with the age of the victim, and that adult female ticks attached to the head or neck, in general, were removed later compared to adult female ticks attached to other parts of the body. This suggests that the site of attachment will influence the probability of early or late detection of an attached tick. Such a difference has, to our knowledge, not been recorded for adult females of *I. ricinus*. An accurate estimation of the duration of tick feeding may be useful when trying to assess whether or not an infection took place and consequently to judge the risk that tick-borne disease symptoms will develop. Duration of tick feeding for adult females and nymphs of *I. ricinus* can be estimated from their scutal and coxal indices [16].

To investigate how different variables influence the risk of developing tick-borne infections, the Tick-Borne Diseases (TBD) STING-study was initiated in 2007 [17,18]. The overall aims of the TBD STING-study are to determine the prevalence and the species of potentially human-pathogenic bacteria and viruses in ticks that have bitten humans. We want to evaluate if parameters such as tick species and stage, the tick’s pathogen content, its attachment site on the host, and its duration of tick feeding, will influence the risk of pathogen transmission and the development of an infection (clinical or serological responses). We have already recorded a mean *Borrelia* prevalence of 26% and a TBEV prevalence of 0.2% among more than 2150 *I. ricinus* ticks that had attached to the skin of persons from regions of Sweden and the Åland Islands [19,20]. The specific aims of the present study were to investigate if there are *i)* any seasonal differences between regions of Sweden and the Åland Islands when people become infested by *I. ricinus*; *ii)* any seasonal differences between the different stages of *I. ricinus* when they bite humans; *iii)* any differences in attachment sites between the stages of *I. ricinus*; *iv)* any stage-related differences in tick feeding durations; and *v)* if the ticks’ attachment sites and the duration of feeding are related to human gender or influenced by the age of the tick-infested persons.

## Methods

### Study design

**Figure 1:**
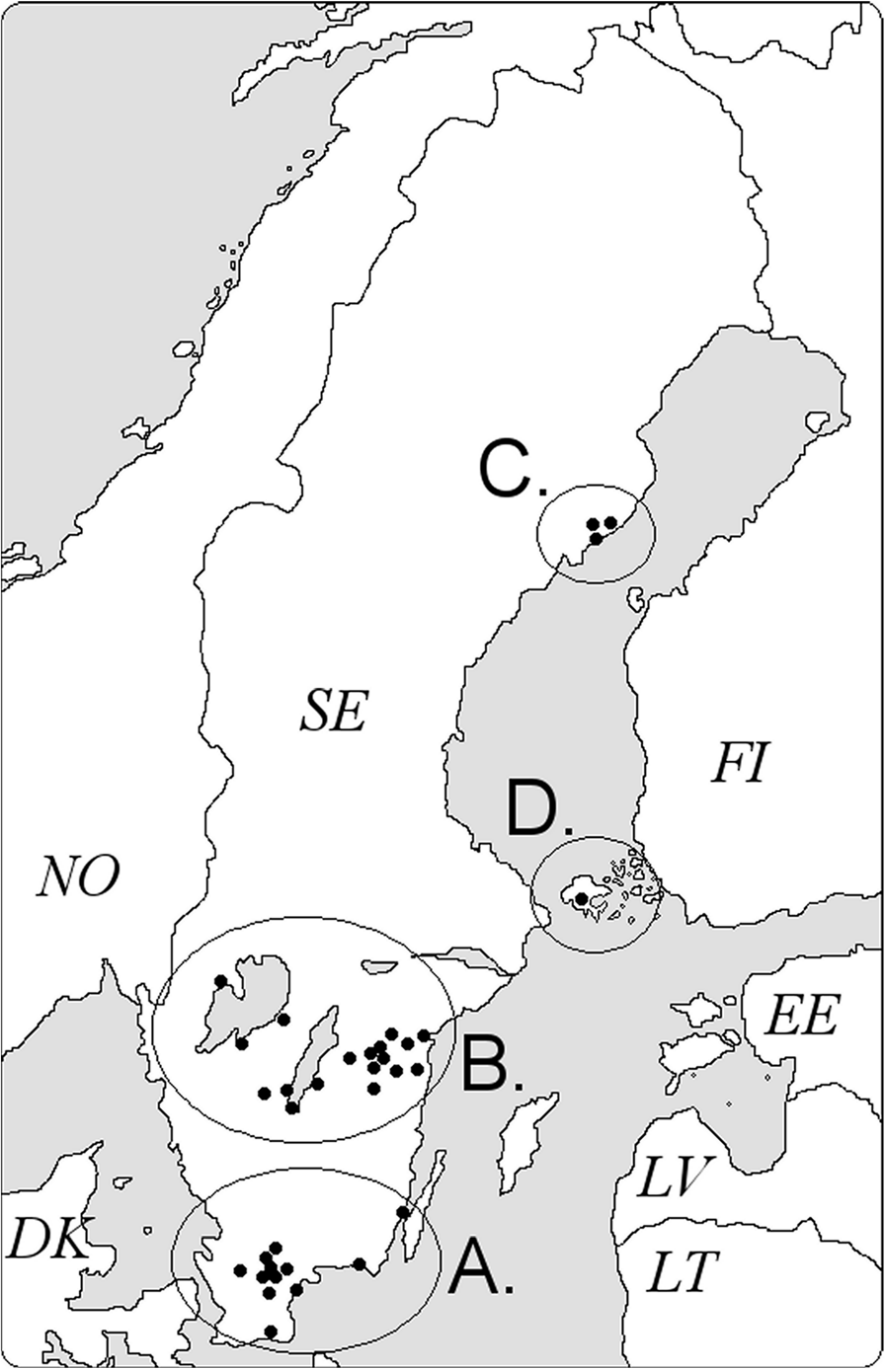
**Location of the 34 primary health care centers (PHCs). (A)** Southernmost Sweden (10 PHCs); **(B)** South Central Sweden (20 PHCs); **(C)** Northern Sweden (3 PHCs); and **(D)** Åland Islands (1 PHCs). SE: Sweden, FI: Finland. Reproduced from [19].

This part of the TBD STING-study was initiated in February of 2008 by advertisements on local television and in newspapers. Persons aged 18 years or older were recruited to the study if they had been bitten recently by a tick. The bitten person was asked to bring the tick(s) to one of 34 primary health care centres (PHCs) located in the regions of Southernmost, South Central, and Northern Sweden, and on the Åland Islands, Finland (Figure 1). At the PHC, the tick-bitten person signed a written consent to participate, donated the removed tick(s), provided a blood sample, completed a questionnaire (Additional file 1), and provided personal data (age and gender). The questionnaire contained questions about the date of detection of the attached tick(s) that led to the study inclusion, probable duration(s) of the tick attachment(s), the attachment location(s) of the tick(s) on the participant’s body, and the estimated number of tick bites the participant had contracted earlier that season.

At a follow-up visit to the PHC three months later, a second blood sample was collected and another questionnaire was completed (Additional file 2). This questionnaire inquired if the participant had had any more tick bites after the first registration. The questionnaire also contained inquiries about the participant’s general health condition during the study period, any symptoms suggestive of tick-borne disease(s) (LB, TBE etc.) and, whether or not, the participant had visited a health care provider due to such symptoms. If the participant had visit a health care provider his/hers medical records were scrutinized in order to determine if he/she was diagnosed with a tick-borne disease. Participants were also asked to collect and donate all ticks that they found attached to their bodies during the study period.

All samples were transported to Linköping University within three days, where the samples were frozen and stored at -70°C until examination.

### Ethical considerations

Before a person was asked to participate in the study, staff of the PHC informed about the general outline and aims, that the participant was allowed to discontinue participation at any time; when so requested, all samples from that person would be discarded. Ethical permission for this study had been approved by the Regional Ethical Review Board, Linköping University (M132-06), and by the local Ethics Committee of the Åland Health Care, 2008-05-23.

### Tick identification and tick feeding duration

Each tick was photographed dorsally and ventrally, using a USB-microscope (Dino-Lite Long AM4013TL, AnMo Electronics Corp., Taiwan) to determine species, life stage, and sex of adults based on [21-23]. To estimate the duration of blood feeding for adult female ticks and nymphs, the scutal index (the ratio of the length of the idiosoma to the width of the scutum) or the coxal index (the ratio of the distance between the basal coxae of the fourth pair of legs to the width of the scutum) were calculated as described by Gray and co-workers [16].

### Statistical analyses

The potential relationships between duration of tick feeding and age classes of participants, between duration of tick feeding and attachment site, and between tick attachment site and gender of participants were evaluated with Chi-square test. This test was also used to compare the proportions of adult female ticks and nymphs that were removed before *versus* after 24 hours of blood feeding. Fisher’s exact test was used when the expected frequency was <5 in at least one of the cells of the contingency table. The Spearman rank correlation test was used to investigate if there was any significant association between the proportions of adult female ticks or nymphs attached > 24 hours and human age classes. Participants were categorized into one of following age classes (years): (19–29), (30–39), (40–49), (50–59), (60–69), (70–79), and (>80). Statistical analyses were performed and graphs were drawn using GraphPad Prism version 5.00 for Windows (GraphPad Software, San Diego, CA). P-values ≤ 0.05 were considered statistically significant.

## Results

### Description of the tick-bitten participants and “their” removed ticks

Between May 2008 and November 2009, 1896 participants attended their first visit to a PHC. Among them, 6.6% (n = 126) were excluded from further analysis due to incomplete answers in the questionnaires. The remaining 1770 participants (648 men and 1122 women) removed and handed in a total of 2110 attached *I. ricinus* ticks (487 adult female ticks [23%], 15 adult male ticks [1%], 1519 nymphs [72%], and 89 larvae [4%]). No other tick species were detected. The proportion of adult female ticks was significantly greater on men (27%) than on women (21%, P < 0.01). In contrast, the proportion of larvae was significantly greater on women (5%) than on men (2%, P < 0.001) (Table 1).

**Table 1:** **Numbers and percentages of the different stages and sexes of *I. ricinus *** **removed from men and women**

**Tick stage**	**No. (%) of ticks removed from**	
	**Men**^**a**^	**Women**^**b**^
Adult female tick	206 (27)	281 (21)
Adult male tick	4 (1)	11 (1)
Nymph	536 (70)	983 (73)
Larva	15 (2)	74 (5)
Total ticks	761 (100)	1349 (100)

^a^n = 648 men.

^b^n = 1122 women.

The median age of the study population was 63 years (range 19–92). Participants aged 60–69 years was the largest age class (35%), while 19–29 year-old participants constituted the smallest group (2%). No significant differences were observed when the proportions of different tick stages were compared with age classes.

### Seasonal tick infestation patterns in the studied regions

**Figure 2:**
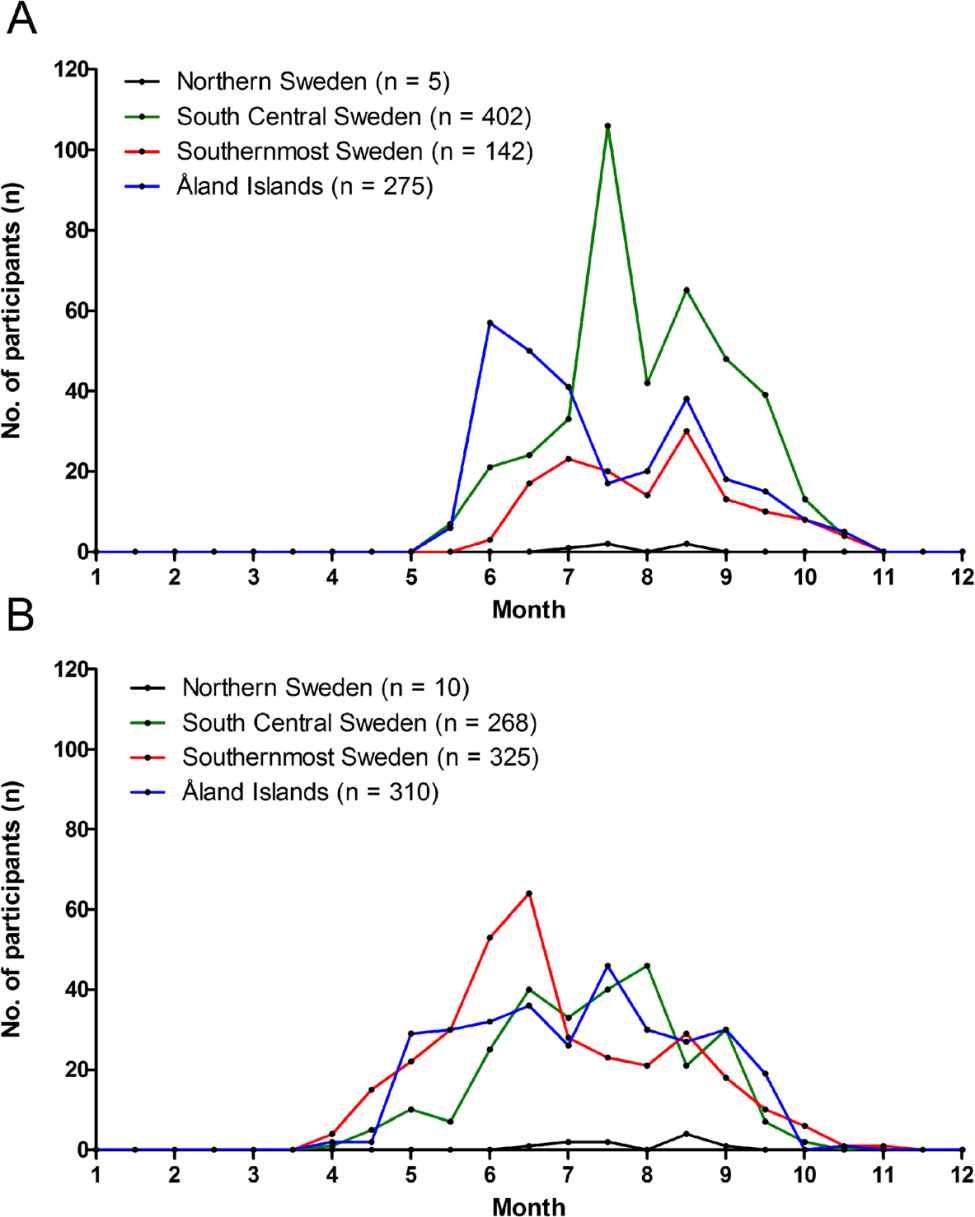
**Monthly distribution of detection of attached ticks by participants in four geographical regions. A**. Study period of 2008 (n = 824), and **B**. Study period of 2009 (n = 913).

Of all participants, 39% were recruited from South Central Sweden (n = 688), 33% from the Åland Islands (n = 590), 27% from Southernmost Sweden (n = 477), and 1% from Northern Sweden (n = 15). Ninety-eight percent of the participants (2008, 824/836; 2009, 913/934) recorded the date when “their” ticks (n = 2056), found attached to the skin, were detected. In 2008, with no regard to the different stages of *I. ricinus*, tick infestations were recorded from mid-May to mid-October (Figure 2A). This was the case for all the studied regions except Northern Sweden where tick infestation on people in 2008 only occurred from early July to mid-August. Ticks collected from the other three regions exhibited a bimodal infestation pattern with a tick infestation depression between mid-July and early-August 2008. In 2009, tick bites were recorded from early April to early November (Figure 2B). This was the case for all the studied regions except Northern Sweden where tick infestation on people in 2009 only occurred from mid-June to early September. Ticks collected from Southernmost Sweden exhibited a bimodal infestation pattern with the first peak in mid-June and the second peak in mid-August. Ticks collected from South Central Sweden and from the Åland Islands exhibited a “dispersed” infestation pattern throughout the tick season of 2009.

### Seasonal infestation of the different stages of *I. ricinus*

**Figure 3:**
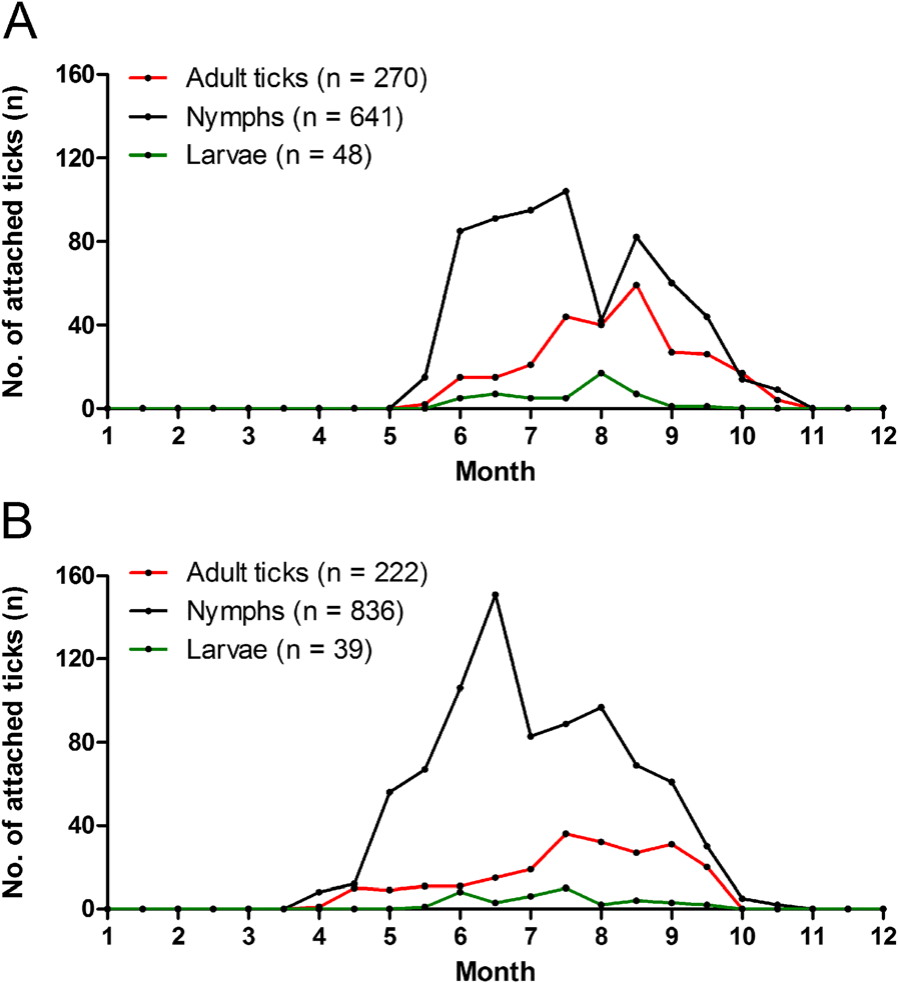
**Monthly distribution of detection of attached *I. ricinus *with respect to stage of development. A**. during 2008 (n = 959), and **B**. during 2009 (n = 1097).

In 2008, with no regard to geographical origin of the ticks, adult ticks and nymphs were detected from mid-May to mid-October (Figure 3A). Larvae were detected from early June to mid-September. The nymphal infestation pattern on humans was bimodal with an infestation depression between mid-July and early August. Adult and larval infestation patterns were unimodal with peaks in mid-August and early August, respectively. In 2009, adult ticks and nymphs were detected from early April and mid-April, respectively, to mid-September and early November, respectively (Figure 3B). Larvae were detected from mid-May to mid-September. The nymphal infestation peaked in mid-June and early August, while peaks of adult and larval infestations took place in mid-July.

### Tick feeding sites on the human body

**Figure 4:**
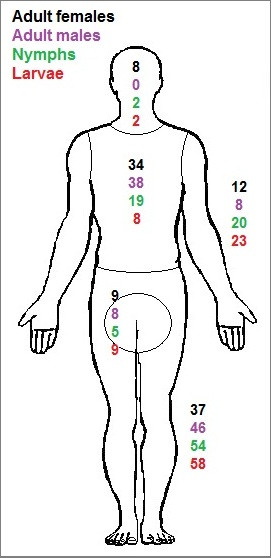
**Anatomical distribution of 1881 removed ticks.** Percentages refer to total number of each tick stage: adult females (n = 459), adult males (n = 13) nymphs (n = 1357), and larvae (n = 52).

Of the 1770 participants, 93% reported the attachment site for a total of 1881 ticks. Among these 1881 *I. ricinus* ticks, a significantly greater proportion of adult female ticks was attached to the skin of the head/neck area (P < 0.001), on the skin of the torso/dorsum area (P < 0.001), and in the groin/genital area (P < 0.01) compared to the proportions of nymphs attached to the corresponding locations (Figure 4). In contrast, greater proportions of nymphs were found on the arms (P < 0.001), and legs (P < 0.001), compared to the proportions of adult female ticks on these extremities. Due to small number of adult male ticks and larvae, they were excluded from the statistical analyses.

**Figure 5:**
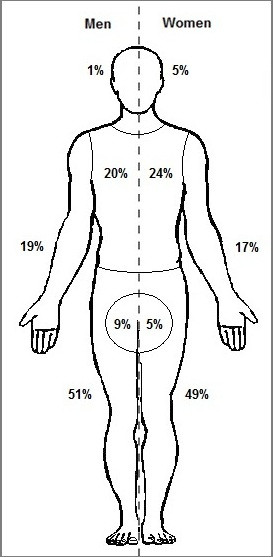
**Anatomical distribution of ticks reported by tick-bitten participants.** Percentages are based on total number of ticks (n = 597) found attached to men (left side) and total number of ticks (n = 1051) found attached to women (right side).

For both men and women, the legs were the major location of tick attachment (51% of 597 ticks on men, and 49% of 1051 ticks on women; Figure 5). This was followed by the torso/dorsum (20% and 24%, respectively) and arms (19% and 17%, respectively). A significantly (P = 0.001) greater proportion of men (9%) than women (5%) recorded ticks attached to the groin/genital area. In contrast, a significantly (P < 0.001) greater proportion of women (5%) than men (1%) recorded ticks attached to the head/neck area. No other significant differences regarding tick attachment sites on men compared to those on women were detected.

No significant differences were found when tick attachment sites were analysed in relation to age classes of the human hosts.

### Duration of tick attachment

Based on the scutal and coxal indices of the ticks [16], we were able to estimate the duration of attachment (= feeding time) for 1710 ticks (410 adult female ticks and 1300 nymphs).

Among the adult female ticks (n = 410), 37% had been attached < 24 hours and 63% > 24 hours (Table 2). When attachment time was analysed in relation to attachment site, a significantly greater proportion (77%) of adult female ticks attached to the skin of the groin/genital area was removed > 24 hours of feeding, compared to the proportion of adult female ticks that was removed > 24 hours from the arms (53%, P < 0.05) or from the legs (59%, P < 0.05). When attachment time, i.e. adult female ticks removed < 24 hours *versus* adult female ticks removed > 24 hours was analysed in relation to participant’s gender, no significant difference was detected (P = 0.08) (Table 3).

**Table 2:** **Attachment durations of *I. ricinus *** **ticks removed from different anatomical sites of human hosts**

**Location of tick bites on people**	**Adult female ticks**		**Nymphs**	
	**No.**	**% > 24 h**	**No.**	**% > 24 h**
Leg	151	59	701	64
Torso/dorsum	140	64	254	65
Arm	51	53	251	54
Groin/genital	39	77	63	67
Head/neck	29	69	31	71
Total	410	37	1300	37

**Table 3:** **Attachment duration (hours) of *I. ricinus *** **ticks feeding on men or women**

**Intervals of attachment in hours**	**No. (%) of adult females removed from**		**No. (%) of nymphs**	
			**removed from**	
	**men**	**women**	**men**	**women**
0-24	55 (33)	99 (41)	143 (32)	343 (40)
24-48	68 (40)	99 (41)	204 (46)	366 (43)
48-72	19 (11)	26 (11)	74 (17)	117 (13)
72-96	18 (11)	10 (4)	20 (4)	29 (3)
> 96	9 (5)	7 (3)	2 (1)	2 (1)
Total	169 (100)	241 (100)	443 (100)	857 (100)

Among nymphs (n = 1300), 37% had been attached< 24 hours and 63% > 24 hours (Table 2). The head/neck area appeared to have the greatest proportion of nymphs (71%) attached > 24 hours, while the arms seemed to have the lowest proportion of nymphs (54%) attached > 24 hours. However, when nymphal data on attachment time was statistically analysed in relation to site of attachment, no significant differences were revealed. When attachment time, i.e. nymphs removed < 24 hours *versus* nymphs removed > 24 hours was analysed in relation to participant’s gender, women removed a significantly greater proportion of nymphs (40%) within 24 hours compared to that of men (32%, P < 0.001) (Table 3).

When the proportion of nymphs attached for > 24 hours was analysed in relation to the age classes of participants, a significant, positive correlation was found (r = 0.89, P < 0.01): The proportion of nymphs attached > 24 hours increased with increasing age class of participants; the age class 30–39 years accounted for the smallest percentage (46%) of nymphs attached > 24 hours, while participants >80 years accounted for the largest percentage (75%) of ticks attached > 24 hours (Table 4). When the proportion of adult female ticks attached for > 24 hours was analysed in relation to the age classes of participants, no significant correlation was found (r = 0.36, P = 0.44).

**Table 4:** **Attachment duration (> 24 hours) of adult female ticks and nymphs of *I. ricinus *** **with respect to age classes of the human hosts**

**Age class(years)**	**Total no. (%) of participants**	**Adult females**^**a**^		**Nymphs**^**a**^	
		**No.**	**% > 24 h**	**No.**	**% > 24 h**
19-29	31 (2)	7	57	20	55
30-39	100 (6)	17	76	76	46
40-49	192 (11)	56	61	127	54
50-59	398 (22)	93	60	303	58
60-69	621 (35)	135	61	469	64
70-79	351 (20)	77	58	253	72
>80	77 (4)	25	84	52	75
Total	1770 (100)	410	62	1300	63

^a^Removed adult female ticks and nymphs where calculation of tick attachment time was possible.

### Calculated time of tick feeding in relation to the participants’ self-estimated time of tick attachment

Calculated times in hours (h) of tick feeding, based on the scutal and coxal indices (intervals 0–24 h, 24–48 h, 48–72 h, and 72–96 h), together with the participants’ self-estimated times of tick attachment were available for 748 ticks. For the calculated interval 0–24 h of tick feeding, participants’ self-estimated the time of tick attachment to 0–91 h (median 15 h, interquartile range [IQR] 7–23 h); for the calculated interval 24–48 h the self-estimated times ranged from 0 to 92 h (median 20 h, IQR 9–33 h); for the interval 48–72 h to 0–91 h (median 20 h, IQR 10–40 h); and for the calculated interval 72–96 h the self-estimated times of tick attachment ranged from 0 to 98 h (median 29 h, IQR 19–50 h).

## Discussion

### Seasonal distribution of infesting ticks

The general seasonal infestation pattern of nymphs during both years was bimodal, with peaks in June-July and August. A similar bimodal seasonal activity pattern of host-seeking nymphs was recorded during most study years in two different field studies in south-central Sweden [7,8], and in one year a unimodal activity pattern was recorded [8]. The depression in nymphal infestation on humans, observed in the present study may, therefore, be a consequence of the depression in nymphal host-seeking activity. The general seasonal infestation pattern of adult ticks during both years was unimodal rather than bimodal. The reason for this is unknown but the adult ticks, compared to nymphs, may have a greater resistance to relative humidity and as a result, they may not exhibit a depression in host-seeking activity pattern during the hottest and driest part of the summer. The numbers of larvae in this study were too low to enable us to draw any conclusions about their seasonal infestation pattern.

In Southernmost Sweden, South Central Sweden, and on the Åland Islands the tick infestation on participants began one month earlier and ended one month later in 2009 compared to 2008 (from early April to early November and from mid-May to mid-October, respectively). According to the Swedish Meteorological and Hydrological Institute (SMHI), in Southernmost Sweden and in South Central Sweden the mean temperature during April 2009 was higher than the mean temperature during April 2008 [24,25]. In addition, the mean temperature for November in these regions was higher in 2009 than in 2008 [26,27]. This may, at least partly, explain why the participants from Southernmost Sweden and South Central Sweden contracted tick bites during an extended time period in 2009 compared to 2008. For both study years in Northern Sweden, the tick infestation on participants lasted for a shorter time period (from early-June to mid-August in 2008 and from mid-June to early September in 2009). The shorter tick infestation period in Northern Sweden may reflect the generally lower abundance of ticks [2], which is a function of a relatively low tick density and relatively low diel and seasonal tick activities, which reflect the generally lower environmental temperature and the shorter growing season in northern Sweden compared to the southern regions. However, to elucidate how temperatures and other climate and weather parameters may influence the seasonal tick infestation pattern in a certain region, frequent sampling and analysis of ticks that have infested humans over multiple seasons are needed. Moreover, the seasonal tick infestation patterns found in this study are influenced not only by the tick’s particular seasonal activity pattern, which may differ among different regions, but also by the varying activities of people, e.g. when people tend to visit tick-infested areas for berry- or mushroom picking or other purposes.

### *Ixodes ricinus* stages and “predilection sites” on humans

In the present study, only few larval ticks were removed. However, this tick stage is considered to be a much less important vector of *B. burgdorferi* s.l. and TBEV infections to humans; the unfed larva is almost never infected with LB-causing bacteria [28], nor with TBEV [29]. The majority of the *I. ricinus* ticks removed were nymphs. This stage is considered to be the most important stage in the transmission of borreliae and TBEV to humans. This is because in nature nymphs are much more numerous than adult ticks, and because nymphs, compared to adult female ticks, are more easily overlooked due to their smaller size and less conspicuous colouration. Even if fewer adult ticks were removed from the participants, adult ticks, compared to immature ticks, are, in general, more often infected with *Borrelia* bacteria [30]. Men removed a greater proportion of adult female ticks compared to women. In contrast, women removed a greater proportion of larval ticks compared to men. However, this does not necessarily imply that a particular tick stage of *I. ricinus* has a preference for a certain gender of *Homo sapiens*. Rather, it may reflect morphological, behavioural and physiological differences between men and women. Such differences, e.g. the usually more hairy skin of men may result in differences between men and women in their capacity to rapidly detect a tick on the skin. Morphological and other differences between men and women and between children and older persons may result in apparently tick-stage-specific “predilection sites” on the human body which may depend on how easy it is for a certain tick stage to find a suitable attachment site. Such “preferred” feeding sites on the host’s body are, most likely, much dependent also on the host’s grooming behaviour.

The majority of the ticks had attached to the legs of the participants. This site of infestation corresponds to the anatomical location where the classical sign of erythema migrans (EM) the hallmark rash of early LB, usually appears. Bennet and co-workers recorded that the most common anatomical localisations of EM among LB patients (n = 118) were the legs (63.6%) followed by torso/dorsum (24.6%), arms (10.2%) and genitalia (1.7%) [31]. These proportions correspond arbitrarily to the anatomical distribution of tick bites recorded by the participants in the present study.

The most commonly infested anatomical location, i.e. legs, is approximately within the same height above the ground where nymphs and adults of *I. ricinus* quest in the vegetation [32]. This suggests that most ticks, searching for an optimal attachment site on a recently encountered human host, will walk only a short vertical distance before they will attach and start to feed. However, the site of tick attachment was related to the stage of *I. ricinus*. Greater proportions of nymphs, compared to adult female ticks, were removed from the extremities of the participants, i.e. from legs and arms, compared to other parts of the body. In contrast, greater proportions of adult female ticks were removed from the skin of the torso/dorsum area, head/neck area, and groin/genital area compared to other parts of the body. Falco and co-workers recorded a similar behaviour, i.e. an apparent “preference” for certain body parts on human hosts by nymphs and adult females of *I. scapularis*[10]. Stage-related differences may, at least partly, be related to the level at which a particular tick stage quests in the vegetation. Adults of *I. ricinus* usually quest at a higher level above ground, compared to nymphs [32]. Thus, the first contact by adult ticks, compared to nymphs, on human hosts should take place further up the human body, closer to the torso/dorsum area and head/neck area. Tick-stage related “preferences” for site of attachment have been observed on other vertebrate hosts. On the white-tailed deer, *Odocoileus virginianus*, the adult *I. scapularis* feed mainly on the anterior dorsal body regions: 87% of adult ticks attached to the ears, head, neck and brisket [33]. These feeding sites by adults of *I. scapularis* correspond arbitrarily to the feeding sites selected by adult females of *I. ricinus* on humans (i.e. torso/dorsum and head/neck areas), found in the present study. On horses, attachment by adult female *I. scapularis* was largely restricted to the under-body areas, which was considered to reflect avoidance of direct sunlight by the ticks [33]. On the European roe deer*,* larvae, nymphs and adult females of *I. ricinus* show high degrees of intrastadial spatial aggregation [34]: larvae aggregate mainly to the forelegs and to the head of roe deer, nymphs aggregate mainly to the head, and adult females aggregate mainly to the neck of roe deer. Stage-specific degrees of tolerance of desiccation may be one among factors, which explain how stage-specific “preferences” for attachment sites have evolved. However, the host’s grooming behaviour and capacity to remove ectoparasites from particular parts of the host’s body should have a great effect on the evolution of feeding sites “preferred” by the ectoparasites.

The site of tick attachment was not influenced by the age of the bitten person. However, women, compared to men, removed a greater proportion of ticks from the head and neck area. Berglund and co-workers [9] found that LB patients bitten on the head or neck more often presented neurologic manifestations compared to LB patients bitten on other parts of the body. This suggests that the site of tick attachment on the skin of the human host may be of particular clinical significance. We also found that men, compared to women, removed a greater proportion of ticks from the groin/genital area. Similar results were recorded in the study of Berglund *et al*. [9]. Consequently, this body region seems to be a “preferred site” for blood-seeking ticks, presumably since here they should be relatively well protected from sunlight, desiccation and host grooming activity.

### Duration of tick attachment

Among adult female ticks and nymphs, 63% were removed later than 24 hours of attachment. When the calculated duration of tick-feeding (based on scutal and coxal indices) were compared to the participants’ self-estimated durations of tick attachment, we found that the tick-bitten persons usually underestimated the duration of tick attachment. A person who removes an attached tick from the skin later than 24 hours of tick attachment is more likely to develop localised and systemic symptoms of tick-borne diseases compared to if the tick is removed earlier [15]. Therefore, it is important to find and remove any tick from the skin as early as possible. For instance, even if the virions of TBEV can be transmitted within 1 hour after tick attachment [12], it is advisable to remove any tick as quickly as possible since the amount of virus particles in the tick salivary gland seems to increase with duration of tick feeding [35]. It is reasonable to assume that the more time a TBEV-infected tick feeds, the higher will be the virus dose transmitted to the host.

The location of the attachment site seemed to influence how soon a tick was detected and, therefore, the duration of tick attachment. Ticks attached to the groin/genital area or to the head/neck area were apparently more difficult to detect than ticks attached to other sites. We did not find any significant differences between adult female ticks and nymphs regarding the time from attachment until the tick was detected and removed. We expected that the larger, adult female ticks would be detected sooner than the smaller and more inconspicuous nymphs. This was reported in a study on *I. ricinus* feeding on humans in Switzerland [36]. The discrepancy between that study and ours, regarding the duration of tick attachment until the nymphs or the adult female ticks were removed, could be due to differences in the participants’ awareness as well as people’s knowledge about tick-infested habitats.

In the present study, the majority of ticks were removed 24–48 hours after the beginning of attachment. Only 5% of the adult female ticks and nymphs were removed after ≥ 72 hours. In transmission experiments using rodents, a high level of *Borrelia* transmission is reached after ≥ 72 hours of tick attachment [13,14,37]. Several studies have shown that few people (6.2-9.0%) become infected with *Borrelia* when they are bitten by a *Borrelia*-infected tick [18,38,39]. One explanation for the low risk of contracting a *Borrelia* infection could be that few ticks (only 5%, in our study) are still attached to the skin after 72 hours.

Older people are likely to have poorer vision and impaired physical sensitivity compared to younger people. This may explain why we found that older participants, compared to younger participants, detected the attached nymphs after a longer attachment time. Similar observations on *I. scapularis* nymphs removed from humans were reported by Falco and co-workers [10]. Moreover, we found that men, compared to women, usually detected any attached tick after a longer duration of tick attachment; this suggests a higher risk of *Borrelia*-transmission to men if they are bitten by *Borrelia*-infected ticks.

## Conclusions

This study describes the tick infestation pattern on humans during two consecutive years. We found seasonal as well as geographical differences in infestation. We also found that the tick attachment site of the human body influenced the time until the tick was discovered and removed. Thus, the site of tick attachment may be of clinical importance. Noteworthy is that most of the ticks (63%) were removed after 24 hours of attachment. Older persons compared to younger ones, and men, compared to women detected “their” ticks slower, i.e. after a more extended tick feeding period, which potentially would be more permissive for pathogen transmission. Information about tick infestation patterns provided by this study should be valuable for the developmental of prophylactic methods against tick infestation and for relevant advice to people on how to avoid or reduce the risk of tick bites.

## Competing interests

The authors have no competing interests.

## Authors’ contributions

PEL, PF, and DN designed the study. PW and PL performed the laboratory analyses. Data analyses and interpretation of data were performed by PW, PL, TGTJ, LF, PF, DN, and PEL. PW and TGTJ wrote the manuscript to which all authors subsequently contributed. The final version of the manuscript was approved by all authors.

## Supplementary Material

Additional file 1 Questionnaire 1. Click here for file

Additional file 2 Questionnaire 2. Click here for file
