# Supplementary material for: Ixodes ricinus ticks removed from humans in Northern Europe: seasonal pattern of infestation, attachment sites and duration of feeding
Source: Parasit Vectors. 2013 Dec 20;6:362. doi: 10.1186/1756-3305-6-362 (PMC3880168; doi:10.1186/1756-3305-6-362)
Supplement: Additional file 1 — Questionnaire 1. [file 1756-3305-6-362-S1.docx]

# **To participants of the STING-study**

## Please answer all questions!

When did you notice that you had been tick-bitten?

Year-Month-Day: ________ ____ ____ Time ________

When do you think you were tick-bitten?

Year-Month-Day: ________ ____ ____ Time ________

Where do you think you were when you were tick-bitten? Please state the

name of the municipality.

_______________________________

What kind of habitat (vegetation type) had you visited?

Lake/Sea  Forest  Garden  Lawn

Other: ___________________________________________________

When was the tick removed?

Year-Month-Day: ________ ____ ____ Time ________

Where on the body was the tick attached? _____________________________________

Did you remove the whole tick? Yes  No  Do not know

Have you had any other tick bites this season? Yes  No  Do not know

If Yes, how many? 1-4  5-9  >10

Have you ever been treated for the tick-borne infection Borrelia?

Yes  No  Do not know  If Yes; Year–Month–Day ________ ____ ____

Did you receive any medicine?

Yes  No  Do not know  If Yes; what kind of medicine did you get? _______________________

Have you ever been treated for “Erythema migrans”?

(Erythema migrans = red ring-like or homogenous expanding rash.)

Yes  No  Do not know  If Yes; Year–Month–Day ________ ____ ____

Did you then receive any medicine to treat the infection?

Yes  No  Do not know  If Yes; what kind?______________________

# **Have you ever been treated for the tick-borne infection “Ehrlichia”**

# **(= Ehrlichiosis, also called “Anaplasma” or anaplasmosis)?**

Yes  No  Do not know  If Yes; Year–Month–Day ________ ____ ____

Did you receive any medicine to cure the Ehrlichia (Anaplasma) infection?

Yes  No  Do not know  If Yes; what kind?_________________________

Have you ever been treated for the tick-borne infection TBE?

(TBE is a viral disease which sometimes causes disease in the central nervous system.)

Yes  No  Do not know  If Yes; Year–Month–Day________ ____ ____

Did you receive any medicine?

Yes  No  Do not know  If Yes; what kind?________________________

Do you have any of the following diseases?

Asthma Yes  No  Do not know

Allergy Yes  No  Do not know

Diabetes Yes  No  Do not know

Tumour-related Yes  No  Do not know

Are you on medication? Yes  No

If Yes; what kind of medicine?

___________________________________________________________________

Do you smoke? Yes  No  Stopped smoking  Year ________

If Yes, how many cigarettes per week? ________

How many years have you smoked? ________

Do you have any pets? Yes  No

Dog Yes  No

Cat Yes  No

Bunny (rabbit) Yes  No

Other: ­­­­­­_______________________________________________

Have you been vaccinated against TBE? Yes  No  Do not know

If Yes; Year-Month-Day________ ____ ____

Have you been vaccinated against Yellow fever? Yes  No  Do not know

If Yes; Year-Month-Day________ ____ ____

Have you been vaccinated against Japanese encephalitis?

Yes  No  Do not know

If Yes; Year-Month-Day________ ____ ____

Thank you for your answers!
