# Supplementary material for: Ixodes ricinus ticks removed from humans in Northern Europe: seasonal pattern of infestation, attachment sites and duration of feeding
Source: Parasit Vectors. 2013 Dec 20;6:362. doi: 10.1186/1756-3305-6-362 (PMC3880168; doi:10.1186/1756-3305-6-362)
Supplement: Additional file 2 — Questionnaire 2. [file 1756-3305-6-362-S2.docx]

Dear STING participant!

Three months have passed since you initiated your participation in the Tick-Borne Diseases STING-study. We previously received blood samples from you and a filled in questionnaire. Now, we need a follow-up blood sample. Therefore, you are requested to visit your primary health care centre at ____________________, week ___, Monday, Tuesday, Wednesday, or Thursday, between _____ and _____ a clock.

If you had any additional tick-bites since your study initiation and if you have collected the ticks in the tube with yellow cork, the please take that tube with you to the blood-sampling.

We would also like to know if you have had any symptoms related to tick-borne diseases during the study period. Please answer the following three questions and write your name, birth date and telephone number on the next page. We might contact you if you reported symptoms. Take this paper to your primary health care centre when you go for the sample-taking.

1) Have you had any additional tick-bites since the first sample-taking?

Yes  No  Do not know

If Yes; when? Year-Month-Day: ________ ____ ____

2) How have you been feeling in general since the first sample-taking?

Have you been feeling good/as usual?

Yes  No  Do not know

If No; please report if you had any of the following symptoms:

Headache Yes  No

Fatigue Yes  No

Fever, 38° or higher Yes  No

Neck pain Yes  No

Loss of appetite Yes  No

Nausea Yes  No

Weight loss Yes  No

Vertigo Yes  No

Concentration difficulties Yes  No

Radiating pain Yes  No

Muscle or joint pain Yes  No

Numbness Yes  No

3) If you reported any symptoms in question 2, did the symptoms appear before or after any additional tick-bites?

Before additional tick-bite Yes  No  Do not know

After additional tick-bite Yes  No  Do not know

4) If you reported any symptoms in question 2, did you visit your primary health care centre due to the symptoms?

Yes  No

5) If you reported any symptoms in question 2, how many days did the symptoms last?

____________________

Thanks for your answers!

Please make sure you answered every question!

Bring this paper to your new sample-taking!

Name: ____________________________________________

Date of birth: _____________________________________________

Telephone number:

Home ___________________________________

Work ___________________________________

Mobile ___________________________________

Best regards

Lotta Lindvall

Forskningssköterska

Infektionskliniken

Universitetssjukhuset

581 85 Linköpings

Tfn xxx/xxx xxxx
